# Metabolomic Profiling of Tomato Root Exudates Induced by *Ralstonia solanacearum* Strains of Different Pathogenicity: Screening for Metabolites Conferring Bacterial Wilt Resistance

**DOI:** 10.4014/jmb.2501.01033

**Published:** 2025-05-26

**Authors:** Zheng Chen, Enquan Lin, Xia Lin, Lianlian Liu, Wangyu Li, Junjie Feng, Bo Liu, Xuefang Zheng, Meichun Chen

**Affiliations:** 1 Institute of Plant Protection, Fujian Academy of Agricultural Sciences, Fuzhou 350013, P.R. China; 2 Institute of Resources, Environment and Soil Fertilizer, Fujian Academy of Agricultural Sciences, Fuzhou 350003, P.R. China; 3 Jinshan college of Fujian Agriculture and Forestry University, Fuzhou 350002, P.R. China

**Keywords:** Bacterial wilt, biofilm formation, metabolome, pathogenicity, *Ralstonia solanacearum*, root exudates

## Abstract

Tomato is one of the most widely cultivated and consumed vegetables in the world, and its production is severely threatened by bacterial wilt. Bacterial wilt caused by *Ralstonia solanacearum*, is one of the most devastating plant diseases. *R. solanacearum* is a complex species with both virulent and avirulent strains. The avirulent strains show high biocontrol activity against bacterial wilt. A metabolomics study was conducted on tomato root exudates induced by *R. solanacearum* of different pathogenicity, and the potential bacterial wilt resistance metabolites were screened. Principal component analysis revealed a clear separation of the metabolic profile between the virulent strain-induced group, the avirulent strain-induced group, and the CK group. Based on the altered abundance in root exudates after *R. solanacearum* induction, the most differential metabolites were selected for further investigation, including citramalic acid, glucuronic acid, alpha-ketoglutaric acid, and malic acid, etc. The plate inhibition assay showed that alpha-ketoglutaric acid (AKG) and malic acid (MA) had a dose-dependent inhibitory effect on *R. solanacearum* (5-20 mg/ml, inhibition circle diameter 14.69-25.08 mm). Crystal violet staining showed that 1-2.5 mg/ml of MA and AKG could significantly inhibit *R. solanacearum* biofilm formation at 24 h (*P* < 0.0001, inhibition rate 66.93-70.43%). In tomato pot experiments against bacterial wilt, the AKG group had 75.36% biocontrol efficacy, and the MA group had 57.97% efficacy 25 days after inoculation. We conclude that AKG and MA play an important role in resistance to bacterial wilt in tomato.

## Introduction

Tomato (*Solanum lycopersicum*) is the world’s second most cultivated vegetable [1] and consumed globally due to its high nutritional value and antioxidant properties [2]. Tomatoes are susceptible to over 200 diseases [3], with soil-borne diseases being a major component [1]. Soil-borne bacterial wilt, caused by *Ralstonia solanacearum*, is considered one of the most devastating plant diseases, resulting in yield losses in many important crops, such as tomato, potato, and tobacco [4-6]. For example, direct yield losses by *R. solanacearum* can be as high as 91% in tomato [5]. *R. solanacearum* naturally infects plants through the roots [7], aggressively colonizes the xylem elements of the vascular system, blocking water transport and causing plants to wilt and die [8]. The management of tomato bacterial wilt using physical, chemical, biological, and cultural methods has been investigated for decades [9]. The use of biological control agents (BCAs) is increasing worldwide and represents a promising strategy for managing soil-borne diseases [10]. Notable BCAs include avirulent strains of *R. solanacearum*, *Pseudomonas* spp., *Bacillus* spp., and *Streptomyces* spp. are Well-known BCAs [9].

*R. solanacearum* is a complex species that has a differentiation of pathogenicity, with a distinction between virulent and avirulent strains. The avirulent strains of *R. solanacearum* can infect, survive, and colonise the host plants but do not cause bacterial wilt [11], while simultaneously preventing the plant from developing bacterial wilt symptoms [12, 13], have been used to effectively reduce the severity of bacterial wilt [14-16]. In our previous work, we isolated a high-purity *R. solanacearum* avirulent strain FJAT-1458 from a tomato host [17], which was shown to have strong biocontrol efficacy against bacterial wilt, which was 100% and 77.45% in pot and field trials, respectively [17, 18]. Further studies have shown that the avirulent strain FJAT1458-RFP could suppress the colonization of the virulent strain in tomato roots, and induce tomato plant resistance to bacterial wilt [19]. However, the mechanisms of biological control of the avirulent strain FJAT-1458 against bacterial wilt are still not fully understood, especially in the field of pathogen-plant interaction. Whether host plants infected by *R. solanacearum* mount an active defense by altering root exudates. It is worth investigating what the differences in tomato root exudates are under *R. solanacearum* of different pathogenicity.

The root system is often referred to as the ‘hidden half ’ of a plant [20]. The importance of root exudates as belowground defense compounds has long been underestimated [21]. Plants invest a substantial fraction of their photosynthesized carbon in root exudates, a collection of low molecular weight compounds released into the rhizosphere [22]. These include sugars and simple polysaccharides, amino acids, organic acids, and phenolic compounds [23]. Root exudation of metabolites is an important mediator of plant interactions with soil microbes [24]. Root exudates play a crucial ecological role in determining the relationship between plants and pathogens, serve as a link between plants, soil, and pathogens, and can influence the occurrence and development of soil-borne diseases [3].

In this study, we investigated the specificity of tomato root exudates induced by different pathogenic strains (virulent strain FJAT-91 and avirulent strain FJAT-1458) using an untargeted metabolomics approach based on GC-TOF-MS and screened for differential metabolites by data analysis. By obtaining these metabolite samples, testing their effects on the inhibition effect and biofilm formation of *R. solanacearum*, and conducting potting experiments with the addition of root exudates, the efficacy of their control of bacterial wilt was confirmed.

Therefore, this work provides a theoretical basis for future research on the mechanism of interaction between plant root exudates and wilt disease, as well as for the prevention and control of soil-borne infections, the restoration of continuous crop barriers, and the creation of sustainable green agriculture.

## Materials and Methods

### Bacterial Stains and Culture Media

The virulent strain FJAT-91 (CGMCC No. 10692) and the avirulent strain FJAT-1458 (CGMCC No. 5111) of *R. solanacearum* used in this study were isolated from bacterial wilt-infected and healthy tomato plants, respectively [19]. Strains FJAT-91 and FJAT-1458 were routinely cultured on TCC medium for 48 h at 30°C. One colony was then suspended on NB medium. After incubation at 170 rpm and 30°C for 24 h, the number of viable bacteria was counted and the initial concentration of FJAT-91 and FJAT-1458 was determined. The bacterial cell density was diluted to 1.5 × 10^8^ CFU/ml.

### Experimental Design and Sample Collection

The tomato (*Solanum lycopersicum*) cultivar “Da-Hong” was used in this study. 4-week-old tomato seedlings were transplanted into 9-cm diameter plastic pots with a capacity of 2,300 ml. Each plant was grown on a commercial potting soil mixture (organic matter ≥45%, N + P_2_O_5_ + K_2_O ≥ 3%, pH 5.5-7.0). Three groups (30 plants each) were used in the pot experiment. FJAT-91 group (inoculated with virulent strain FJAT-91), FJAT-1458 group (inoculated with avirulent strain FJAT-1458), and the control group (sterile water). Each experiment was conducted in a greenhouse at 30 ± 2°C, 80% relative humidity, and 12 h of light and 12 h of darkness. After plant root injury treatment, 200 ml of FJAT-91 and FJAT-1458 fermentation broth, diluted to 1.5 × 10^8^ CFU/ml, was poured into the soil around the plants. The control group received 200 ml of sterile water. Rhizosphere soil samples were collected after the appearance of bacterial wilt in plants belonging to the FJAT-91 group. Ten tomato plants were randomly selected simultaneously from each group. After lyophilization, rhizosphere samples were collected and pre-frozen at -80°C in a refrigerator for 48 h. After collection, the lyophilized powder was ground through a 40 mesh filter, packaged, and stored.

### GC-TOF-MS Analyss and Data Preprocessing

A 5 ml tube was filled with 400 ± 10 mg of soil sample, and 1 ml of the pre-cold extraction mixture (methanol: dH_2_O = 3:1, v/v) and 1 ml of ethyl acetate with internal standard (adonitol, 0.5 mg/ml stock) were added. After 30 sec of vortexing and 4 min of ball milling at 40 Hz, the samples were sonicated and centrifuged. After evaporation in a vacuum concentrator, 40 μl Methoxyamination hydrochloride (20 mg/ml in pyridine) was added and then incubated at 80°C for 30 min, then derivatized with 60 μl of BSTFA regent (1% TMCS, v/v) at 70°C for 1.5 h. The samples were cooled gradually to room temperature, and 5 μl of FAMEs (in chloroform) were added to the QC sample.

GC-TOF-MS analysis was performed using a Shimadzu GC-2020 gas chromatograph coupled with a time-of-flight mass spectrometer. The system used a DB-5MS capillary column. The helium gas flow rate through the column was 1 ml min^-1^ , and the front inlet purge flow was 3 ml min^-1^. The initial temperature was held at 50°C for 1 min, then raised to 310°C at a rate of 8°C min^-1^, then held at 310°C for 11.5 min. The ion source, injection and transfer line temperatures were 200, 280 and 280°C, respectively. The energy was -70 eV in electron impact mode. Mass spectrometry data were acquired in full scan mode with the m/z range of 50-500 at a rate of 12.5 spectra per second after a solvent delay of 7.2 min. A 1 μl aliquot of sample was injected in splitless mode. Data preprocessing and annotation: Raw data analysis, including peak extraction, baseline adjustment, deconvolution, alignment and integration, was completed using Chroma TOF (V 4.3x, LECO) software and the LECO-Fiehn Rtx5 database was used for metabolite identification by matching the mass spectrum and retention index. Finally, the peaks detected in less than half of QC samples or RSD > 30% in QC samples were removed [25].

The selection criteria for the main differential metabolites were P-value in the Student’s *t*-test < 0.05, variable importance in projection (VIP) ≥ 1.0, and absolute fold change (FC) <0.67 or >1.5. The identified metabolites were annotated using the KEGG compound database (http://www.kegg.jp/kegg/compound/), and the annotated metabolites were then mapped to the KEGG pathway database (http://www.kegg.jp/kegg/pathway.html).

### Effects of Differential Metabolites on the Inhibition of *R. solanacearum*

Metabolomic analysis of the root exudate samples identified several differential metabolites. In order to accurately uncover potential antimicrobial metabolites, the fold change (FC) value was used as a key indicator, and reference was made to the antimicrobial activity and the metabolic pathway in which they are located, as reported in the available literature. Using this approach, several metabolites with antimicrobial potential were screened, including α-ketoglutaric acid, malic acid, phosphorylethanolamine, glucuronic acid, fructose, and citramalic acid.

These compounds were individually tested for in vitro antimicrobial activity against *R. solanacearum* using the filter paper disc agar diffusion method [26]. *R. solanacearum* FJAT-91 was cultured in NB medium at 30°C for 48 h. The bacterial culture suspension was homogeneously dispersed in semi-solid NA medium at 40-50°C, at a controlled concentration of 10^7^ CFU/ml, and poured into Petri dishes (90 mm) coated with solid NA medium. After solidification, four 7-mm-diameter wells were made in each plate, and 100 μl of each metabolite was injected into each well at a concentration of 5, 15 and 20 mg/ml. The plates were then incubated at 30°C for 48 h and the diameter of the inhibition zone was measured. All experiments were performed in triplicate.

The minimum inhibitory concentration (MIC) of the KGA and MA was determined using the sterile 96-well polystyrene microtiter plates, following the method of Wiegand *et al*. [27] with some modifications. The final concentrations of KGA and MA were set at 5.000, 2.500, 1.2500, 0.6250, 0.3125, 0.1563, 0.0781, 0.0391, 0.0195, 0.0098, and 0.0049 mg/ml, respectively. Meanwhile, the final bacterial concentration of the FJAT-91 fermentation broth was maintained at 5 × 10^5^ CFU/ml. Samples were then incubated at 32°C for 24 and 48 h, then removed for observation and their OD_600_ values were measured to determine the MIC.

### Effect of Differential Metabolites on *R. solanacearum* Biofilm Formation

With some minor modifications, a modified microtiter plate assay [28] was used to quantify the production of *R. solanacearum* biofilms. Briefly, a sterile 24-well polystyrene microtiter plate was inoculated with *R. solanacearum* FJAT-91 (1 × 10^8^ CFU/ml), the bacterial film was fixed with methanol, stained with crystal violet, the bound dye was released with 33% glacial acetic acid, and the optical density (OD) of the solution was measured at 570 nm using a microplate spectrophotometer. Five replicates and seven different concentrations (2.5, 1, 0.5, 0.2, 0.1, 0.005, 0 mg/ml) were used in to set up of each assay. The biofilm inhibition percentage was calculated using the formula:

Biofilm inhibition (%) =((A_570_ of biofilm in control- A_570_ of biofilm in treatment /A_570_ of biofilm in control)) × 100%.

### Effect of Differential Metabolites on Tomato Bacterial Wilt on Pot Experiment

The pot experiments were performed according to the approach of Zhou X, *et al*. [29], with some modifications. Briefly, 4-week-old tomato seedlings were transplanted into the plastic pots containing a commercial potting soil mixture (organic matter ≥45%, N + P_2_O_5_ + K_2_O ≥ 3%, pH 5.5-7.0). The plastic pots were 9 cm in diameter with a capacity of 2,300 ml (the actual volume of soil filled is 1,600 ml), each pot contained 4 seedlings. In this experiment, three sets of 30 seedlings each were used, and all the tomatoes were naturally inoculated with the *R. solanacearum* FJAT-91. In the MA treatment group, the soil surface of each seedling received 25 ml of MA at a dosage of 5 mg/ml every 72 h, in the AKG treatment group, the soil surface of each seedling received 25 ml of AKG at a dosage of 5 mg/ml every 72 h, while the control group was simply inoculated with FJAT-91. In the MA and AKG treatment groups, the soil surface of each seedling received 25 ml of MA (or AKG) at a dosage of 5 mg/ml. The expected concentration of MA and AKG in the soil was 5 mg/ml × 100 ml/ 1,600 ml = 0.3125 mg/ml, higher than the MIC (24 h). Each experiment was conducted in a greenhouse at 30 ± 2°C, 80% relative humidity, and 12 h of light and 12 h of darkness. The typical symptoms of bacterial wilt were evaluated according to a disease index (di) on a scale of 0 to 4. The DIR, DSI and biocontrol efficacy were calculated as follows:

The disease incidence rate (DIR) = (number of diseased plants/ total number of inspected plants) × 100%.

The disease severity index (DSI) = (∑(number of diseased plants in the index × di)/(total number of treated plants × highest di)) × 100%.

The biocontrol efficacy =((DSI of control - DSI of MA (or AKG) treated group))/DSI of control ×100% [30].

## Results

### Metabolic Profiling of Tomato Root Exudates

Potted tomatoes were inoculated with *R. solanacearum* of different pathogenicity (virulent strain FJAT-91 and avirulent strain FJAT-1458), FJAT-91 treated tomatoes showed wilt symptoms, while FJAT-1458 tomatoes remained healthy, and root soil was collected for non-targeted metabolomics analysis.

**Fig. 1:**
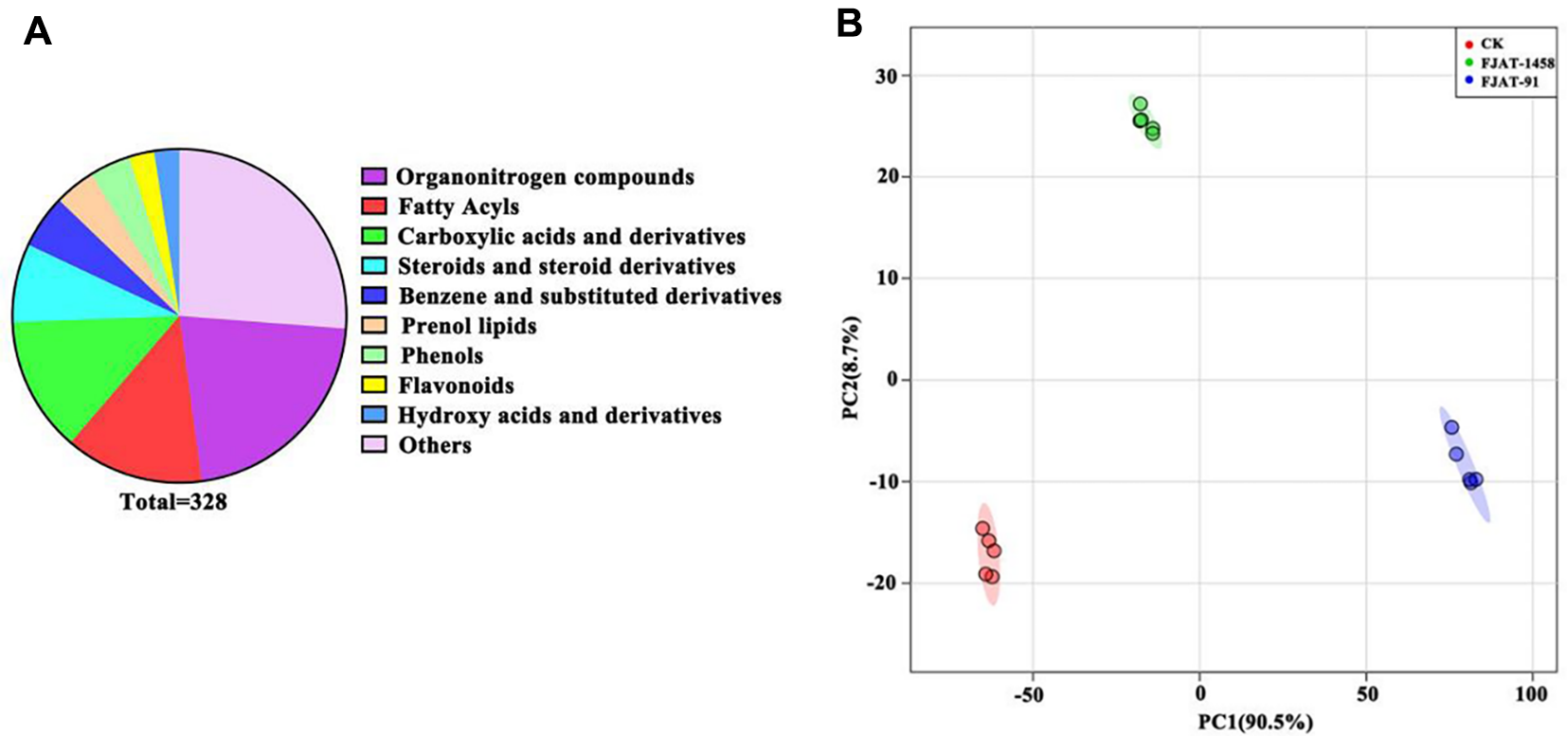
Metabolomic analysis of tomato root exudates with different pathogenic strains of *Ralstonia solanacearum* (FJAT-91, *Ralstonia solanacearum* virulent strain; FJAT-1458, *Ralstonia solanacearum* avirulent strain). (**A**) Number of different types of identified metabolites; (**B**) Principal component analysis score plot of metabolites.

The tomato root exudate samples were subjected to GC-TOF-MS metabolomics analysis, and a total of 328 chemicals were identified (Fig. 1A), including 71 organonitrogen compounds, 44 fatty acyls, 43 carboxylic acids and derivatives, 25 steroids and steroid derivatives, 17 benzene and substituted derivatives, 13 prenol lipids, 13 phenols, 8 flavonoids, 8 hydroxy acids and derivatives.

As shown in the principal component analysis (PCA), components PC1 and PC2 accounted for 90.5% and 8.7%of the total variance, respectively (Fig. 1B). A distinction was observed between the treated (FJAT-91, FJAT-1458) and control (CK) samples. In addition, samples from the FJAT-91 and FJAT-1458 groups also segregated.

### Metabolomic Changes in the Root Exudates of Bacterial Wilt and Heathly Tomato

Several metabolites were selected based on VIP >1.0 and fold change ≥1.50 or ≤0.67 between samples, several metabolites were selected. The top 20 components with the highest absolute values of the logarithm of the multiplicity of differences were selected after metabolites were analyzed for differences between treatment groups.

**Fig. 2:**
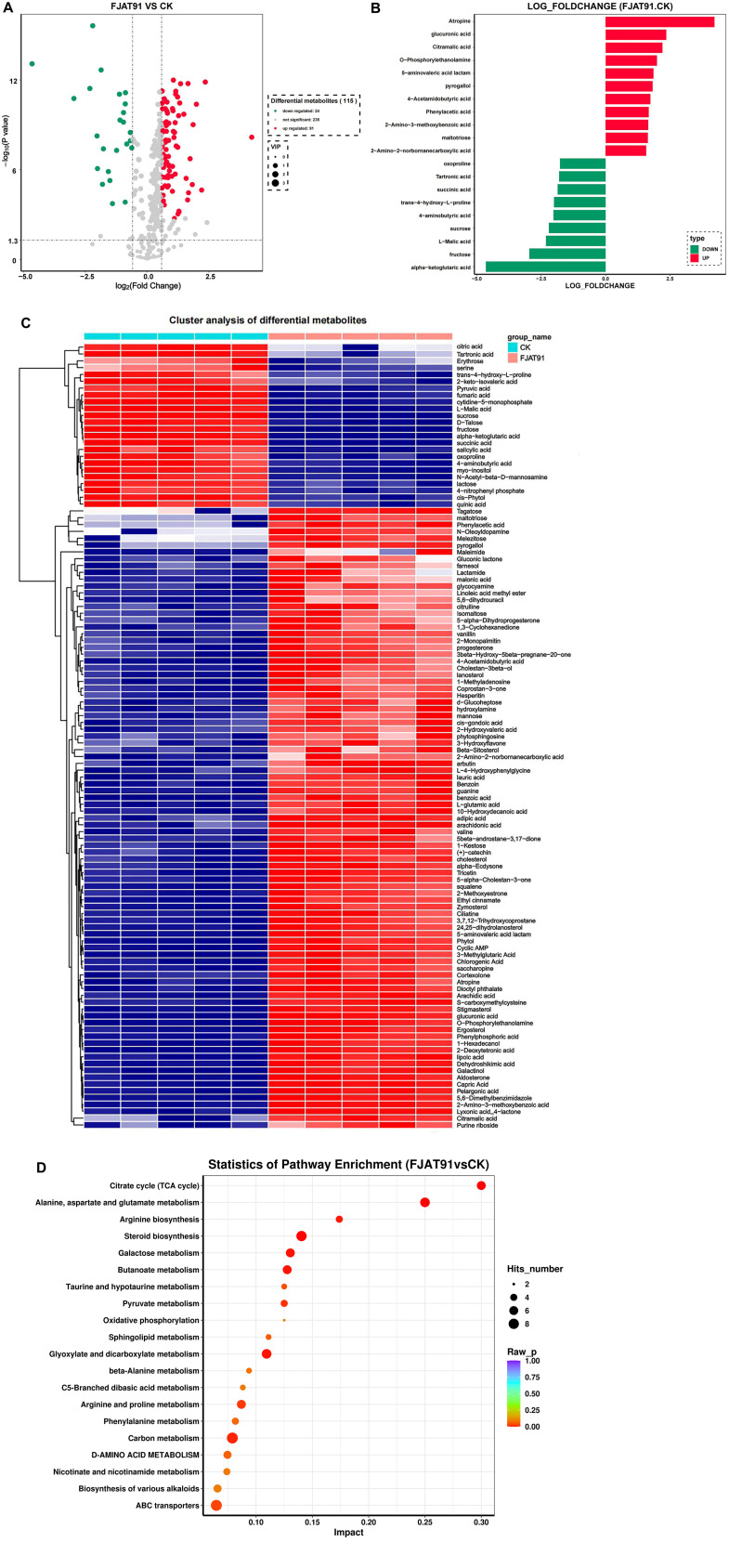
Metabolomic Changes in Root Exudates of bacterial wilt and Heathly Tomato (FJAT-91, *Ralstonia solanacearum* virulent strain; CK, the control). (**A**) Volcano plot analysis of differential metabolites of FJAT-91 vs CK ; (**B**) Histogram of differential metabolites in FJAT-91 vs CK ; (**C**) Heatmap of hierarchical cluster analysis for FJAT-91 and CK; (**D**) KEGG pathway enrichment analysis of differentially accumulated metabolites.

There were 115 differential metabolites between the FJAT-91 and CK groups, of which 91 were up-regulated and 24 were down-regulated (Fig. 2A). Up-regulated metabolites included atropine, glucuronic acid, citramalic acid, O-phosphorylethanolamine, and 5-aminovaleric acid lactam, etc. Down-regulated substances included alpha-ketoglutaric acid, fructose, malic acid, sucrose, 4-aminobutyric acid, etc. (Fig. 2B). Namely, *R. solanacearum* infection inhibited the accumulation of alpha-ketoglutaric acid, malic acid, fructose, etc., but increased the content of atropine, glucuronic acid, citramalic acid, etc.

To analyze the metabolic pathways of healthy and diseased tomato root exudates, we selected the top 20 enriched KEGG pathways (Fig. 1D). Several pathways, such as citrate cycle (TCA cycle); alanine, aspartate and glutamate metabolism; arginine biosynthesis; steroid biosynthesis; galactose metabolism and butanoate metabolism show significant differences in the FJAT-91 vs CK samples.

### Metabolomic Changes in Tomato Root Exudates Induced by *R. solanacearum* Strains of Different Pathogenicity

**Fig. 3:**
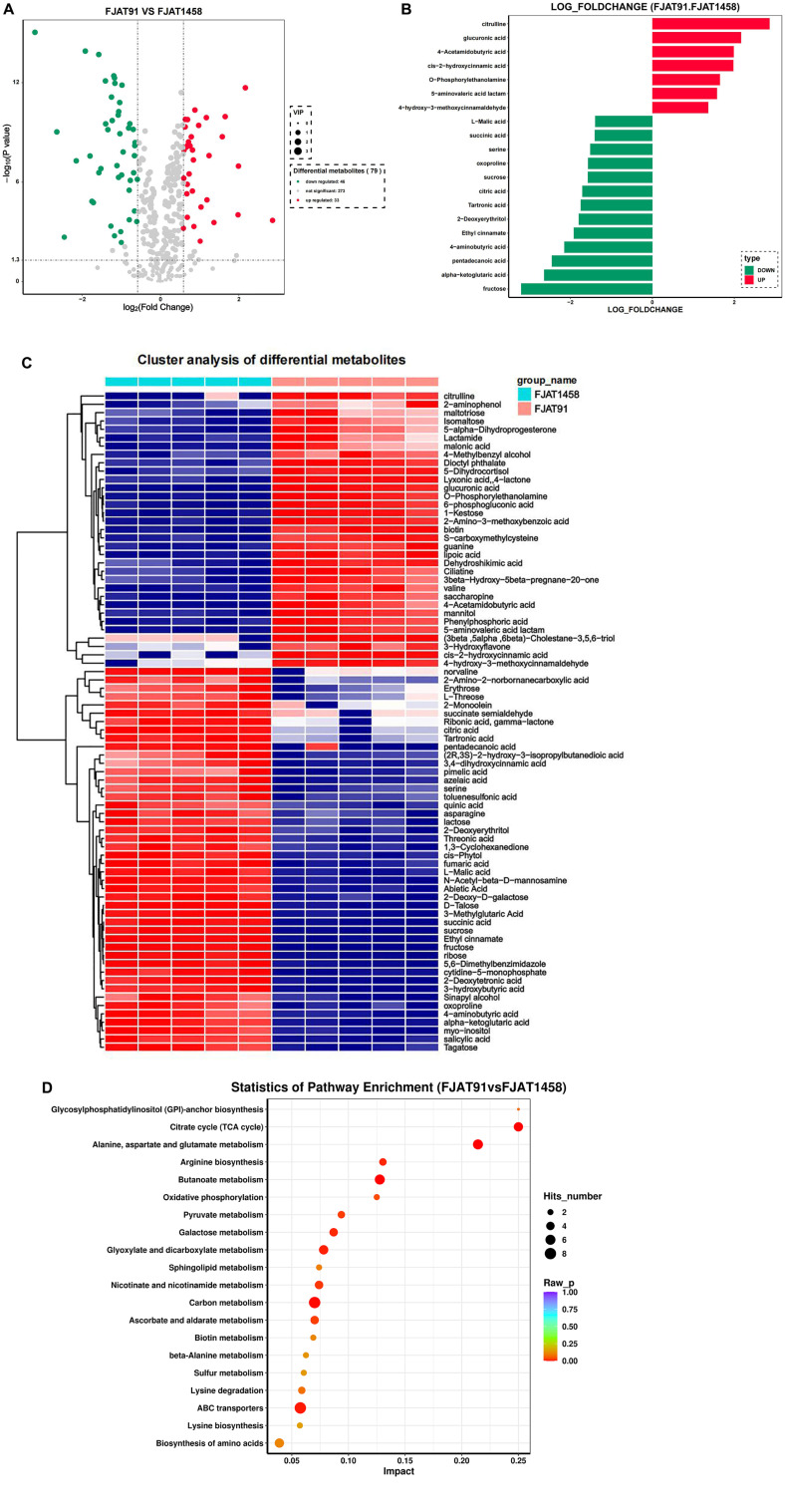
Metabolomic changes in tomato root exudates induced by *Ralstonia solanacearum* strains of different pathogenicity. (**A**) Volcano plot analysis of FJAT-91 vs FJAT-1458; (**B**) Histogram of differential metabolites in FJAT-91 vs FJAT-1458; (**C**) Heatmap of hierarchical cluster analysis for FJAT-91 and FJAT-1458; (**D**) KEGG pathway enrichment analysis of differentially accumulated metabolites.

There were 79 differential metabolites between the FJAT-91 and FJAT-1458 groups, of which 33 were up-regulated and 46 were down-regulated (Fig. 3A). The up-regulated metabolites included citramalic acid, glucuronic acid, 4-acetamidobutyric acid, cis-2-hydroxycinnamic acid, O-phosphorylethanolamine, 5-aminovaleric acid lactam, and 4-hydroxy-3-methoxycinnamaldehyde. Down-regulated substances included fructose, alpha-ketoglutaric acid, pentadecanoic acid, 4-aminobutyric acid, ethyl cinnamate, 2-deoxyerythritol, tartronic acid, citric acid, sucrose, oxoproline, serine, succinic acid and malic acid (Fig. 3B).

To analyze the metabolic pathways of root exudates from tomato plants induced by variable pathogenicity of *R. solanacearum*. The top 20 enriched KEGG pathways are shown in Fig. 3D. The KEGG functional annotation of these differential metabolites highlighted several metabolic pathways that underwent notable changes, including the following: glycosylphosphatidylinositol (GPI)-anchor biosynthesis; citrate cycle (TCA cycle); alanine, aspartate and glutamate metabolism; arginine biosynthesis; butanoate metabolism.

### Effects of Differential Root Exudates on the Inhibition of *R. solanacearum*

We performed an analysis of the metabolites shown in Figs. 2B and 3B, focusing on their antimicrobial activities and associated metabolic pathways as documented in the literature. To identify potential antimicrobial metabolites, we employed a screening approach using the fold change (FC) values of differential metabolites as a key indicator. Among the screened metabolites, several showed promising antimicrobial potential, including α-ketoglutaric acid, malic acid, phosphorylethanolamine, glucuronic acid, fructose and citramalic acid.

The antimicrobial efficacy of these six compounds against *Ralstonia solanacearum* are shown in Table 1. The results showed that AKG (α-Ketoglutaric acid ) has the strongest inhibitory activity, while MA (malic acid) also shows relatively strong activity. Glucuronic acid and phosphorylethanolamine have weak inhibitory activity and are only effective at higher concentrations (> 10 mg/ml). Citramalic acid and fructose show no inhibitory activity in the concentration range tested.

**Fig. 4:**
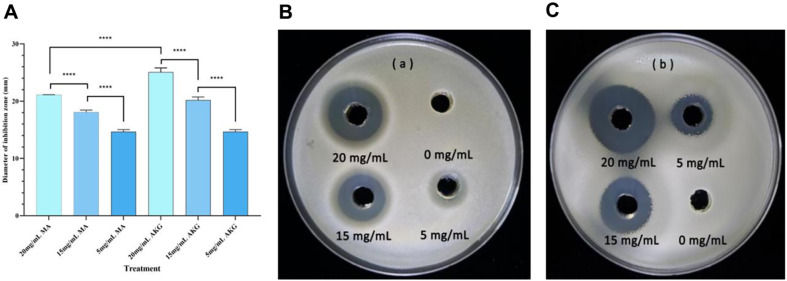
Inhibitory effect of MA and AKG against *Ralstonia solanacearum* FJAT-91. (**A**) Analysis of variance of the size of the circle of inhibition of MA and AKG at different concentrations; (**B**) MA (malic acid); (**C**) AKG (alpha-ketoglutaric acid). ****P < 0.0001.

The results showed that when AKG (alpha-ketoglutaric acid) was added at concentrations of 5, 15, and 20 mg/ml, the diameter of the inhibition circle was 14.69 ± 0.36, 20.20 ± 0.54, and 25.08 ± 0.74 mm, respectively (Table 1, Fig. 4B); when MA (malic acid) was added, the diameter of the inhibition circle was 14.69 ± 0.36, 18.09 ± 0.36, and 21.11 ± 0. 02 mm, respectively (Table 1, Fig. 4C).

Analysis of variance (ANOVA) showed that the results for different concentration treatments were highly significantly different (*P* < 0.0001) (Fig. 4A). At a concentration of 20 mg/ml, the inhibitory effect of AKG was significantly higher than that of MA (*P* < 0.0001). It can be concluded that MA and AKG had an inhibitory effect on *R. solanacearum* with a dose-dependent effect, ranging from 5 mg/ml, 15 mg/ml and 20 mg/ml.

The minimum inhibitory concentration (MIC) of AKG against *R. solanacearum* FJAT-91 was 0.3125 mg/ml at 24 h and remained the same at 48 h. Similarly, the MIC of MA was 0.1563 mg/ml at 24 h and increased to 0.3125 mg/ml at 48 h. These results indicate the effective concentration ranges of AKG and MA in inhibiting the growth of *R. solanacearum* FJAT-91 over different time periods.

### Effects of MA and AKG on *R. solanacearum* Biofilm Formation

**Fig. 5:**
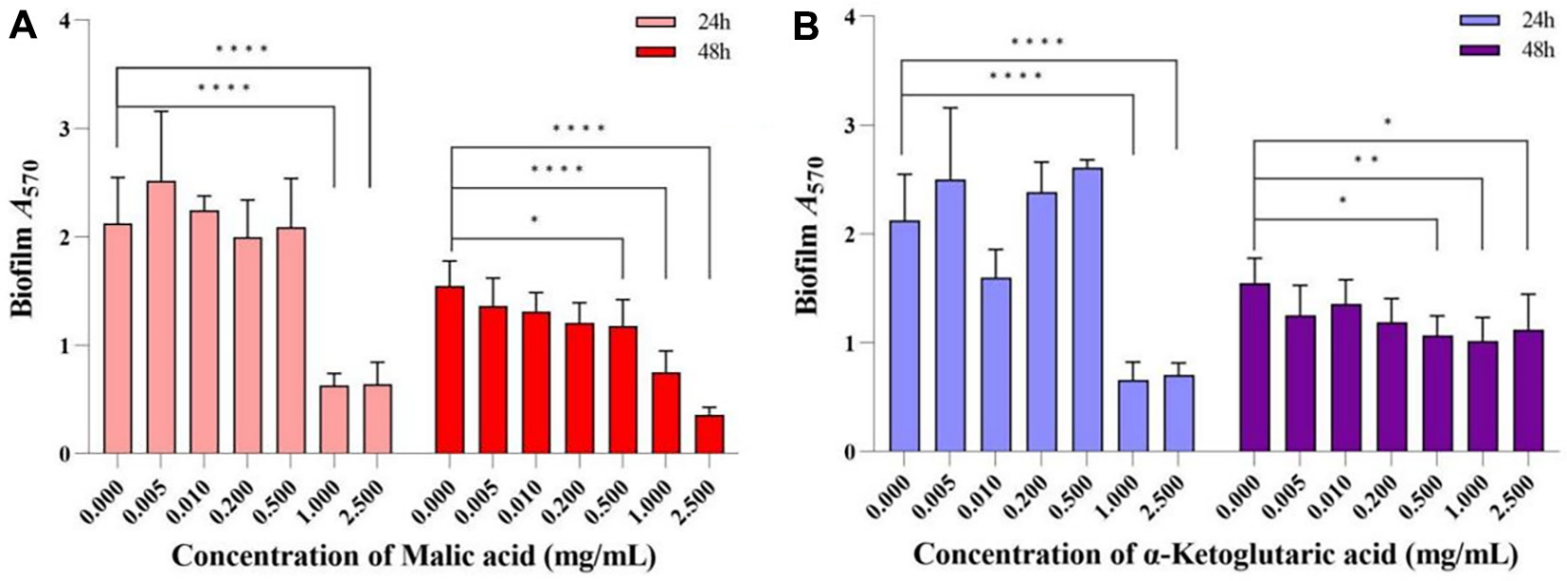
Effect of MA (**A**) and AKG (**B**) on biofilm formation of *Ralstonia solanacearum* at 24 and 48 h. ****P < 0.0001, **P < 0.01, *P < 0.05.

The effects of MA and AKG treatments on *R. solanacearum* biofilm formation were determined by crystal violet staining. The results showed that after 24 h of incubation, MA and AKG at concentrations of 1 mg/ml and 2.5 mg/ml were able to reduce the amount of *R. solanacearum* biofilm formation very significantly (*P* < 0.0001), the biofilm inhibition rate was 66.93-70.43% (Fig. 5A and 5B). After 48 h of incubation, MA at concentrations of 1 mg/ml and 2.5 mg/ml still significantly reduced the amount of *R. solanacearum* biofilm formation (*P* < 0.0001), the biofilm inhibition rate was 51.48-76.95% (Fig. 5A), and AKG at concentrations of 1 mg/ml also reduced the amount of biofilm formation (*P* < 0.01), with a biofilm inhibition rate of 34.16% (Fig. 5B).

### Biocontrol Activity of MA and AKG on Tomato Pot Experiment against Bacterial Wilt

**Fig. 6:**
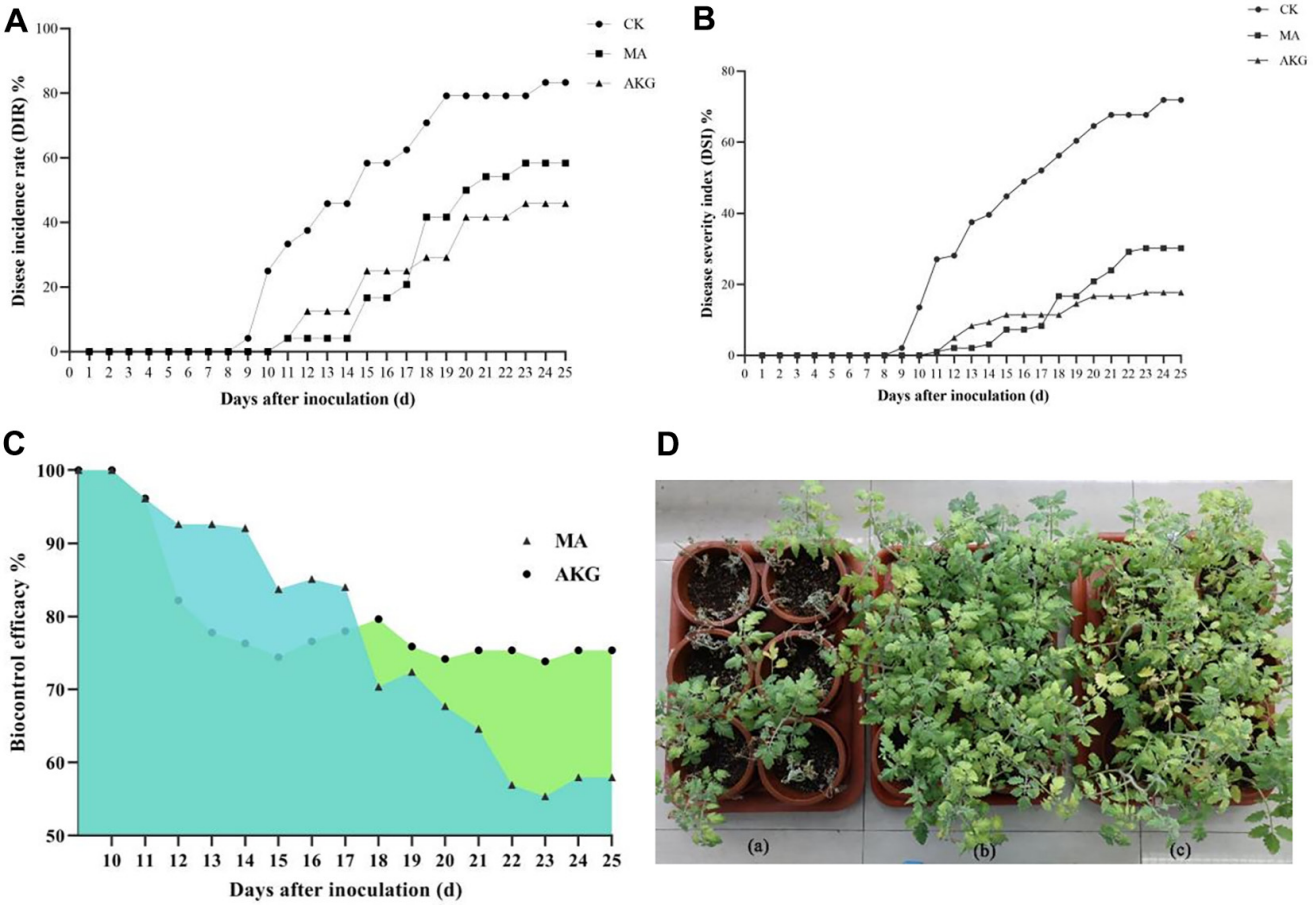
Biocontrol activity of MA and AKG on tomato pot experiment against bacterial wilt. (**A**) Disease incidence rate (DIR%); (**B**) disease severity index (DSI%); (**C**) biocontrol efficacy; (**D**) seedlings treated with MA (or AKG) for 25 days post inoculation a. CK (inoculated with FJAT-91); b. MA treatment group; c. AKG treatment group.

The biocontrol activity of MA and AKG against tomato bacterial wilt was evaluated by the pot experiment. At 9 days after inoculation with *R. solanacearum* cell suspensions, the tomato plants in the control treatment showed wilt symptoms, whereas the plants treated with MA and AKG remained healthy. Disease incidence rate (DIR) in the negative control treatment reached 83.33% at 25 days after inoculation (Fig. 6A). bacterial wilt symptoms in tomato plants were delayed approximately by about 2 days when the MA and AKG were applied.

At 17 days post inoculation, the disease severity index (DSI) in the MA treatment group (8.33%) and AKG treatment group (11.46%) was much lower than that in the negative control group (52.08%), achieving biocontrol efficiencies of 84.01% and 78.00%, respectively (Fig. 6B). In addition, the AKG treatment group continued to show 75.36% biocontrol efficacy against tomato bacterial wilt at 25 days post inoculation, and the MA treatment group showed 57.97% biocontrol efficacy (Fig. 6C and 6D). The biocontrol activity of MA treatments was superior to AKG at 12-17 d post inoculation, while AKG showed better control at 18-25 d post inoculation (Fig. 6C).

## Disussion

Pathogen-infested plants produce a large number of secondary metabolites, some of which are resistance metabolites that disrupt the structure of the pathogen and inhibit its growth and reproduction [31]. In recent years, with the development of mass spectrometry and metabolomics, screening for disease resistance chemicals in plants using non-targeted metabolomics has shown great promise for understanding the mechanisms underlying plant disease resistance and for developing innovative approaches to disease prevention and control. Tang W, *et al*.[32] applied non-target metabolomics to potato spindle tuber viroid (PSTVd)-infected and control SC-5, and identified xanthohumol and echinocystic acid as inhibitors of PSTVd titer. Mareya CR, *et al*. [33] used untargeted metabolomics on two sorghum cultivars with different disease tolerance infected with *Burkholderia andropogonis*, monitored metabolic changes, and identified salicylic acid, jasmonic acid, and zeatin as significant defense-related biomarker metabolites. In this study, an untargeted metabolomics was performed on tomato root exudates induced by the virulent and avirulent strain of *R. solanacearum* and control to identify potential anti-bacterial wilt metabolites.

Plant root exudates can serve as chemical signal substances to regulate the activity of soil pathogenic microorganisms and induce the occurrence of soil-borne diseases by altering the soil microecological environment [34]. Plant root exudates often undergo significant changes in quantity and composition under biotic stress [35]. In this study, R. solanacearum-induced changes in the profile of tomato root exudates were observed. A total of 328 root exudates were detected, and principal component analysis (PCA) revealed a clear separation of the global metabolic profile between the FJAT-91-induced group, the FJAT-1458-induced group, and the CK group. Further analysis showed that 115 differentially expressed metabolites were found between the FJAT-91 and CK samples. Up-regulated metabolites included atropine, glucuronic acid, citramalic acid, etc. While down-regulated substances included AKG, fructose, sucrose, MA etc. Similar results were previously reported by Wen, *et al*. [36], who also observed a high abundance of *R. solanacearum* down-regulated rhizosphere soil metabolites such as sucrose, fructose. Metabolomic changes were also observed in tomato root exudates induced by *R. solanacearum* with different pathogenicity. 79 differential metabolites were found between the FJAT-91 and FJAT-1458 groups. Based on the altered abundance in root exudates after *R. solanacearum* induction, the most significantly differential metabolites were selected for further investigation, including citramalic acid, glucuronic acid, AKG, MA, atropine and 4-aminobutyric acid, etc.

*R. solanacearum* has a complex pathogenicity system with a large number of virulence factors, including type IV pilus system, extracellular polysaccharide (EPS), lipopolysaccharide (LPS), and the type III secretion system. Among them, EPS, LPS and extracellular proteins are also important components of the biofilm formed by *R. solanacearum* during the colonization process of the root surface and roots [37]. Thus, biofilm formation plays a critical role in bacterial survival and adaptation. For pathogenic bacteria, biofilm formation is a critical factor in their ability to invade host inter-roots [38]. A large number of researchers have devoted their efforts to uncovering plant resistance to biofilm formation by R. Solanacearum, discovering substances such as coumarin [39], hydroxycoumarins [40], daphnetin [41], esculetin [40], umbelliferone [40], resveratrol [40] and protocatechualdehyde [40]. This study showed that MA and AKG from tomato root exudates also had significant activity in inhibiting biofilm formation of *R. solanacearum*. The crystal violet staining results showed that after 24 h of incubation, MA and AKG at concentrations of 1- 2.5 mg/ml were able to reduce the amount of *R. solanacearum* biofilm formation very significantly (*P* < 0.0001), with the biofilm inhibition rate reaching 66.93-70.43%.

Organic acids are major components of root exudates, which are critical for rhizosphere ecology, nutrient acquisition, and plant-microbe interactions [42]. In recent years, bactericidal, fungicidal, and nematodocidal activities of AKG, citric, succinic and other organic Acids have been demonstrated [43]. AKG is an intermediate products of the TCA cycle and plays an important role in amino acid and protein metabolism [44]. It has fungicidal and nematocidal effects in plant protection against phytopathogens [45] and is also an important growth regulator in plant [46]. AKG can suppress the growth of the phytopathogenic fungus *Fusarium napiforme* and the nematode *Ditylenchus destructor* [45]. AKG could accelerate of nitrogen assimilating enzymes and the antioxidant system under arsenate toxicity, and enhance *Solanum melongena* L. Growth [46]. Pyridine (a derivative of AKG) and hydrazine are considered to be antiviral and antibacterial compounds as well as the plant growth regulators [47]. MA is also an important intermediate in the TCA cycle, has antimicrobial activity against *Listeria monocytogenes* [48], *Listeria monocytogenes* [49], *Salmonella Typhimurium* [49], *Salmonella enteritidis* [48], and *Escherichia coli* [48] etc. In addition to direct bacterial inhibition, MA is also able to protect against plant diseases by recruiting bioprophilic bacteria. Rudrappa T, *et al*. [50] showed that MA secreted from roots of Arabidopsis (*Arabidopsis thaliana*) selectively signals and recruits the beneficial rhizobacterium *Bacillus subtilis* FB17 in a dose-dependent manner. Yuan, *et al*. [51] reported that MA in banana root exudates promoted the colonization of the beneficial *Bacillus amyloliquefaciens* NJN-6, protecting banana from soil-borne pathogens. De Weert, *et al*. [52] showed that MA is essential for the colonization and chemotaxis of *Pseudomonas fluorescent* WCS365 in tomato roots. The levels of MA were decreased in *Pseudomonas syringae*-infected kiwifruit [53], Our results also showed that MA and AKG were down-regulated in *R. solanacearum*-infected tomato root exudates. Plant defense heavily drains TCA cycle-generated energy and intermediates to support the costly defense-related metabolic pathways [54]. The reduction in the levels of MA and AKG in the tomato root exudates following *R. solanacearum* infection may be related to this mechanism. Our results showed that AKG and MA play an important role in resistance to tomato bacterial wilt. The plate inhibition assay also showed that AKG and MA had an inhibitory effect on *R. solanacearum* with a dose-dependent effect, ranging from 5-20 mg/ml, and the diameter of the inhibition circle was 14.69-25.08 mm. The tomato pot experiment also showed that the AKG treatment group still had 75.36% biocontrol efficacy against tomato bacterial wilt 25 days after inoculation, and the MA treatment group had 57.97% biocontrol efficacy.

We conclude that AKG and MA play an important role in resistance to *R. solanacearum*. Our knowledge of the role of plant root exudates in plant-pathogen interactions is enhanced by the results of the current study, which provides a theoretical basis for future investigations into the mechanism of interaction between plant root exudates and wilt disease. Our study also provides a statistical basis for the breeding of bacterial wilt-resistant tomato cultivars and the development of herbal chemical preparations to prevent wilt.

## Figures and Tables

**Table 1:** Diameter of the circle of inhibition of different root exduates against *Ralstonia solanacearum*.

Component	Inhibitory zone diameter (mm, $\bar{x}$ ± SD)		
	5 mg/ml	15 mg/ml	20 mg/ml
α-Ketoglutaric acid	14.69 ± 0.36	20.20 ± 0.54	25.08 ± 0.74
Malic acid	14.69 ± 0.36	18.09 ± 0.36	21.11 ± 0. 02
Phosphorylethanolamine	-	13.06 ± 0.13	15.33 ± 0.40
Glucuronic acid	-	12.76 ± 0.53	14.60 ± 0.58
Citramalic acid	-	-	-
Fructose	-	-	-

Data presented as mean ± SD (n=3).

– Not detected.
